# The Relationship Between Family Functioning and Internalizing Problems in Chinese Adolescents: A Moderated Mediation Model

**DOI:** 10.3389/fpsyg.2021.644222

**Published:** 2021-03-24

**Authors:** Qiuying Wang, Siya Peng, Xinli Chi

**Affiliations:** ^1^Virtual-Physical Arts Research Center, Educational Science Research Institute of Shenzhen, Shenzhen, China; ^2^School of Psychology, Shenzhen University, Shenzhen, China

**Keywords:** internalizing problems, family functioning, positive youth development, migrant children, Chinese adolescents

## Abstract

Research has consistently found that poor family functioning is a risk factor for adolescents' internalizing problems. However, studies of the mediating and moderating mechanisms underlying this relation are insufficient. In this study, we explore the association between family functioning and adolescents' internalizing problems by testing the mediating roles of positive youth development (PYD) attributes and the moderating role of migrant status. A large cross-sectional sample of 11,865 Chinese adolescents (mean age = 14.45 years, standard deviation = 1.55 years) were used to measure internalizing problems, family functioning, PYD, migrant status, and other demographic information. After controlling for covariates (age, gender, grade, and socioeconomic status), the results revealed that PYD mediated the relation between family functioning and internalizing problems. Moreover, migrant status moderated the relationship between family functioning and internalizing problems. Specifically, the effects of family functioning on internalizing problems were stronger among local-born adolescents than among migrant adolescents. The findings indicate that improving family functioning and PYD attributes may be promising approaches to prevent/reduce adolescent internalizing problems.

## Introduction

Adolescence is a period of rapid physical, psychological, and social development. In this period, problem behaviors of adolescents can frequently occur, such as internalizing problems. Internalizing problems is generally considered a branch of psychopathology that involves emotional or mood disorders (Graber and Sontag, 2009). Currently, studies have indicated that teenagers are the group with the highest incidence of internalizing problems (Shek, 2002a; Graber and Sontag, 2009; Jamnik and Dilalla, 2019), which is harmful to their healthy development (Rosenfield et al., 2005), including school maladaptation (Jankowska et al., 2015), low social functioning (Ohtani et al., 2015), sleep disturbance (Pieters et al., 2015), substance use (Miettunen et al., 2014), and even self-harm and suicidal behavior (Lee et al., 2006). Despite extensive research exploring the influencing factors of internalizing problems among adolescents in the Western countries, studies are inadequate in China. China has the second largest youth population in the world, and the mental health problems of adolescents are increasingly serious (Xiong et al., 2017). Thus, there is an urgent need to identify the relevant factors and underlying mechanisms of adolescent internalizing problems in order to develop effective prevention/intervention programs.

According to the ecological model, the family environment is the most direct and predominant environment of an individual's development (Bronfenbrenner, 1979). Based on this model, a series of studies have explored the relationships between family factors and adolescents' internalizing problems (Ha et al., 2009; Martin et al., 2010; Ma et al., 2013). Among these factors, family functioning plays an especially important role in adolescent development. Generally, family functioning is regarded as the ability of the family to perform its functions, such as meeting the physical and emotional needs of its members (Dickstein, 2002; Eichelsheim, 2011). Increasing evidence suggests that family functioning is significantly associated with adolescents' internalizing problems (Shek, 2002a; Ma et al., 2013), such as anxiety and depression (Sheeber et al., 1997, 2001; Guberman and Manassis, 2011; Stark et al., 2012). For example, previous studies have found that adolescents with strong family cohesion will develop less anxiety and fewer withdrawal behaviors (Roosa et al., 1996; Johnson et al., 2001). In contrast, poor family relationships (e.g., parent–child conflicts and conflicts between parents) increase the risk of depressive symptoms among adolescents (Formoso et al., 2000; Liu et al., 2011; Claire et al., 2013).

Although previous studies indicated that family functioning was negatively associated with internalizing problems, the underlying mediating mechanism (i.e., how family functioning influences internalizing problems) and moderating mechanism (i.e., when family functioning is related to internalizing problems, or the different influence of family functioning on internalizing problems in different groups) remain unclear, particularly with regard to Chinese adolescents. Therefore, in this study, we constructed a moderated mediation model to test the mediating role of positive youth development (PYD) and the moderating role of migrant status on the relationship between family functioning and internalizing problems in Chinese adolescents.

### The Mediating Role of PYD

To comprehensively study adolescents' positive development, the concept of PYD was brought forward, consisting 15 positive developmental qualities (i.e., bonding, resilience, social competence, recognition for positive behavior, emotional competence, cognitive competence, behavioral competence, moral competence, self-determination, self-efficacy, clear and positive identity, belief in the future, prosocial involvement, prosocial norms, and spirituality), and emphasizing the development potential of teenagers to cope with their growing environment. An increasing number of studies have suggested that the characteristics of PYD play an important role in the healthy development of adolescents (Chang and Zhang, 2013). The development assets framework emphasizes the joint effects of external assets (e.g., environmental characteristics) and internal assets (e.g., psychological resources) on adolescent development (Benson et al., 2007). Moreover, the model has also stressed that external resources may impact adolescent behavioral performance through internal resources (Chang and Zhang, 2013). Empirical studies have supported this view. For example, previous studies found that healthy family functioning (e.g., good family communication) is correlated with higher level of positive development (e.g., greater self-esteem and resilience), as well as a lower likelihood of anxiety and depression in adolescents (Russell et al., 2000; Stark et al., 2012; Yee and Sulaiman, 2017). PYD attributes (e.g., resilience, prosocial behavior, etc.) were significantly associated with less internalizing problems among adolescence such as adolescent depression (Catalano et al., 2002; Reivich et al., 2013; Shek and Sun, 2013; Leung and Shek, 2015; Leung et al., 2017). Additionally, favorable family functioning (e.g., frequent communication, family harmony, a good parent–child relationship) can promote PYD features such as resilience, self-efficacy, socioemotional competence, and prosocial behaviors in adolescents (Renzaho et al., 2013; Cigala et al., 2014; Reitz et al., 2014; Yee and Sulaiman, 2017), which may further predict the lower likelihood of internalizing problems (e.g., depression; Reivich et al., 2013; Wang and Saudino, 2015; Rocchino et al., 2017; Yee and Sulaiman, 2017). Based on the development assets framework and previous studies, PYD is expected to act as an important factor that mediates the relationships between family functioning and internalizing problems (Hypothesis 1).

### The Moderating Role of Migrant Status

According to person–context interaction theory, the interaction between the individual and the environment determines individual development (Magnusson and Stattin, 1998; Lerner et al., 2006), which indicates that the negative effect of unhealthy family functioning on adolescents may vary from individual to individual (Claire et al., 2013). Several studies have suggested that migrant status may be a risk factor exacerbating the impact of a poor family environment (e.g., family functioning and parent–child relationship) on psychological well-being (e.g., life satisfaction), which may be attributed to environmental adaptation pressure and acculturation stress (Cheung, 2013; Wang et al., 2014; Shi et al., 2019). For instance, researchers have reported that poor family functioning had a more negative effect on life satisfaction in migrant children than in local-born children (Hou et al., 2009; Yuan et al., 2019). However, several other studies took the opposite view, arguing that despite the acculturative stress, migrant students had better academic performance and higher levels on a psychological well-being index than their local-born counterparts, which may be attributed to increased resilience from migration (Fan et al., 2009; García-Coll and Marks, 2012; Serap et al., 2018). For example, previous research found that strict parental discipline had a greater negative impact on problem behaviors in local-born children but not in immigrant children (Hackett et al., 1991). These findings indicate that whether migrant status plays a risk-buffering or risk-intensifying role in the relationship between family environmental factors and adolescent mental health remains inconsistent and requires further research. Based on person–context interaction theory and previous studies, we hypothesized that migrant status moderates the link between family functioning and internalizing problems (Hypothesis 2), as well as the link between family functioning and PYD (Hypothesis 3).

### The Present Study

**Figure 1:**
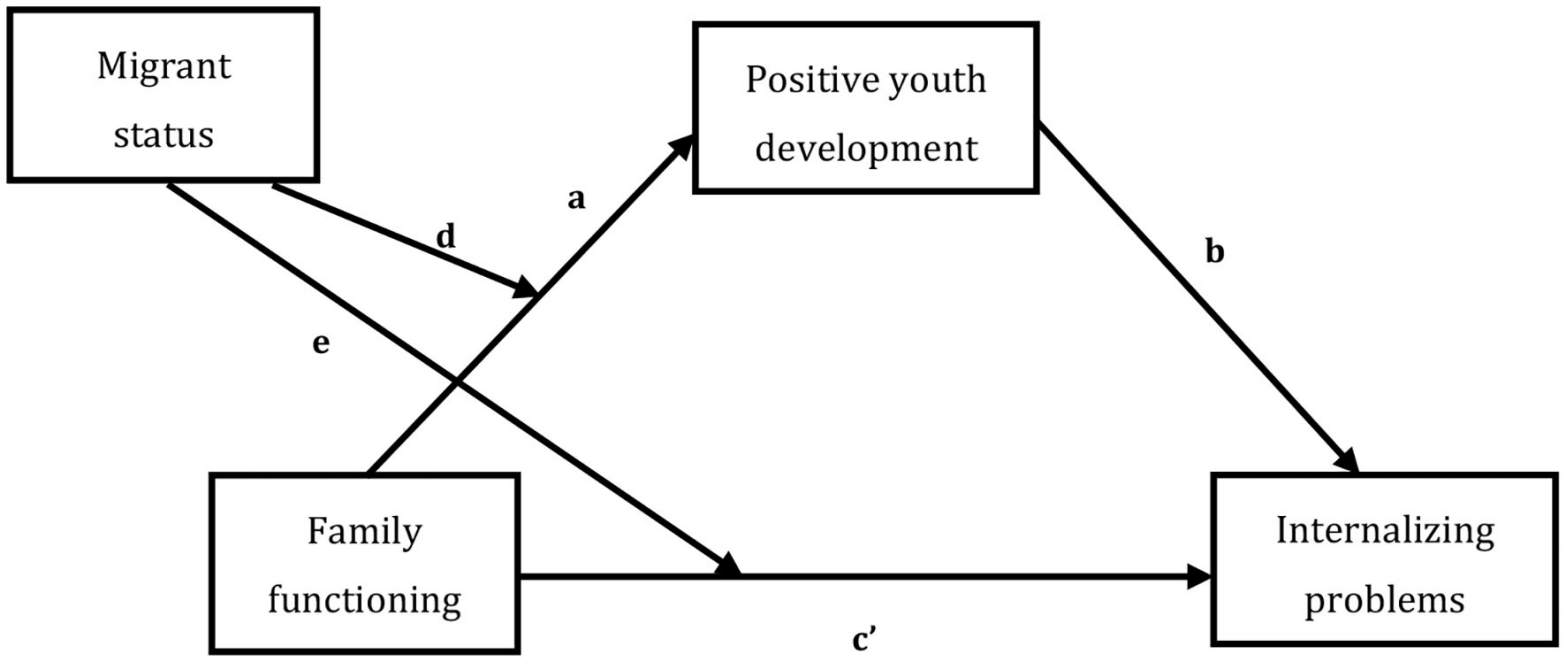
The moderated mediation model. a = the relation between family functioning and positive youth development; b = the relation between positive youth development and internalizing problems; c′ = the relation between family functioning and internalizing problems; d = the moderating effect of migrant/local-born adolescents on the relation between family functioning and positive youth development. e = the moderating effect of migrant/local-born adolescents on the relation between family functioning and internalizing problems.

In summary, based on ecological theory, the development assets framework and person–context interaction theory, we constructed a moderated mediation model (Figure 1) to explore the effects of family functioning, PYD, and migrant status on adolescents' internalization of problems. Specifically, this study attempted to examine the mediating effects of PYD and migrant status on the association between family functioning and internalizing problems in adolescents. We expect that the results of the study can provide suggestions for the prevention of and intervention in adolescent problem behaviors.

## Methods

### Participants and Sampling

Participants were recruited from high schools in the city of Shenzhen in southern China. Shenzhen is a typical migrant city in China. Up to 2018, there were ~390 middle and high schools in Shenzhen, with 448,000 students enrolled in school, of which nearly 40% were migrant students (Anonymous, 2018). Thus, it is appropriate to select Shenzhen for immigration-related research. With the assistance of the Shenzhen Educational Science Research Institute, an investigation was conducted using a large population for multistage random sampling. Based on the total number of schools in Shenzhen and our sampling ratio, we invited 70 public middle and high schools (seven schools in each of 10 districts) to participate in the study, and a total of 67 schools accepted the invitation. In each school, 200 students who agreed and had consent from their guardians to participate in the study were randomly recruited.

Initially, 12,244 adolescents participated, and the valid data from 11,865 were ultimately studied. The mean age of the adolescents was 14.45 years [standard deviation (SD) = 1.55 years, range = 12–18 years], including 5,883 boys (49.6%) and 5,982 girls (50.4%). With regard to the grade levels, 4,250 students (35.8%) were in the seventh grade, 3,498 students (29.5%) were in the eighth grade, 1,159 students (9.8%) were in the ninth grade, 1,356 students (11.4%) were in the 10th grade, 841 students (7.1%) were in the 11th grade, and 761 students (6.4%) were in the 12th grade. The sample included 9,344 local-born adolescents (78.8%) and 2,521 migrant adolescents (21.2%); all the migrant adolescents (i.e., internal migration within China) came from cities other than Shenzhen, and they had similar ethnic and cultural backgrounds (i.e., collectivist culture) as the adolescents born in Shenzhen. The average duration of their migration to Shenzhen was 7.82 years (SD = 3.538 years).

### Procedure

The study was performed in classroom settings at participants' schools using written questionnaires. Each school received official approval and obtained the informed consent of participants and their guardians in advance. Recruitment and data collection procedures were approved by the Human Research Ethics Committee of Shenzhen University. Twenty trained research assistants took charge of the data collection, with teams of two to three research assistants responsible for 40 to 50 adolescents. Participants were randomly assigned to different classrooms. To maximize the effectiveness of self-reporting, we conducted pilot testing with standardized instructions and assured participants that their answers would be anonymous and confidential.

### Measures

#### Demographic Information

Demographic information consisted of age, gender, grade, migrant status, parental educational levels, and family monthly income. Migrant status was determined by the yes/no question, “Are you a migrant emigrating to Shenzhen from other cities?” The family socioeconomic status (SES) was assessed via parental educational level and family monthly income. Specifically, there were four options for parental educational level: (a) middle school graduation or lower, (b) high school or junior college graduation, (c) bachelor's degree graduation, and (d) master's degree graduation or higher. With regard to family monthly income, there were five available options: (a) 2,000–3,999 CNY per month, (b) 4,000–5,999 CNY per month, (c) 6,000–7,999 CNY per month, (d) 8,000–9,999 CNY per month, and (e) more than 10,000 CNY per month. Following the suggestion of Kraus et al. (2009), we standardized parental educational level and family monthly income and then summed them for the indicator of family SES.

#### Family Functioning

Family functioning was assessed by the Family Function core scale, which is a revised version of the Chinese Family Assessment Instrument (C-FAI) adapted by Shek (2002b). The C-FAI is suitable for adolescent participants with a Chinese cultural background and has five dimensions: communication, mutuality, conflict, parental concern, and parental control. For the current study, we selected three subscales (nine items) of the C-FAI: communication (three items, e.g., “My parents and I often have conversations”), mutuality (three items, e.g., “My family lives in harmony”), and conflict (three items, e.g., “We have a lot of conflict,” reverse scoring). Items were answered on a five-point scale ranging from 1 to 5 (1 = very dissimilar, 5 = very similar). The mean value of each subscale was used as an indicator of family functioning. The higher the score, the better the family's functioning. Previous studies have confirmed the reliability and validity of the C-FAI (e.g., Leung and Shek, 2015). For the current study, Cronbach α for the questionnaire was 0.87.

#### Positive Youth Development

PYD was assessed using the Chinese Positive Youth Development Scale (Shek et al., 2007). Because Chinese teenagers generally have no religious belief, we chose 14 dimensions of the scale other than spirituality: bonding (three items), resilience (three items), social competence (three items), recognition for positive behavior (three items), emotional competence (three items), cognitive competence (three items), behavioral competence (three items), moral competence (three items), self-determination (three items), self-efficacy (two items), clear and positive identity (three items), belief in the future (three items), prosocial involvement (three items), and prosocial norms (three items). A sample item was “If I'm sad, I can express my emotions properly.” Items were answered on a 6-point scale ranging from 1 to 6 (1 = strongly disagree, 6 = strongly agree), and the average of the total score was used as an indicator of adolescent positive development. For the current study, Cronbach α for the questionnaire was 0.98.

#### Internalizing Problems

To measure participants' internalization of problems, we selected the evaluation scale for internalizing problems by teenagers, which is a subscale of the Evaluation Scale for Risk Behavior of Teenagers established by Bai (2007). The scale is mainly divided into 4 dimensions and 21 items: anxiety (six items, e.g., “I seem to be easily frightened”), depression (nine items, e.g., “I am considered a quiet, perverse person”), neuroticism (three items, e.g., “I often feel my heart beating fast”), and withdrawal (three items, e.g., “I dare not speak loudly in front of many people, and I always hide away quietly”). Items were answered on a 5-point scale ranging from 1 to 5 (1 = never, 5 = always). The higher the participant's score, the more serious their internalization of problems. For the current study, Cronbach α for this questionnaire was 0.96.

### Statistical Analyses

After entering the data into a computer, we used SPSS 21.0 software for data analysis. The steps are as follows: common method biases analysis was performed first; second, descriptive statistics of the main variables and Pearson correlation analysis were conducted; third, a non-parametric test (Mann–Whitney *U*-test) was done to determine the differences with migrant status for all factors; fourth, the PROCESS plug-in developed by Hayes was used, with models four and eight selected to test the mediation model and the moderated mediation model, respectively (Hayes, 2013). The confidence interval (CI) was 95%, and there were 5,000 bootstrap samples. That the 95% CI does not contain zero suggests a significant conditional indirect effect. Previous studies found that age, gender, grade, and family SES were significantly correlated with adolescents internalizing problems (Leadbeater et al., 1999; Bradley and Corwyn, 2002; Yu et al., 2017), so we controlled for gender, age, and SES as covariates.

## Results

### Testing of Common Method Biases

Because of the self-report method of data collection, there may be a common method bias. Therefore, the subjects were asked to complete the questionnaire anonymously, and some items were controlled by reverse questions. Further, Harman's single-factor analysis was used to test the common method variance effect (Xiong et al., 2012). The results showed that the variance interpretation rate of the first common factor was 33.70%, less than the critical standard of 40%, indicating that the influence of the common method bias in this study was relatively minor.

### Preliminary Analyses

First, the data were analyzed using descriptive statistics, including the mean and SD of family functioning (mean = 4.17, SD = 0.75), PYD (mean = 5.05, SD = 0.74), and internalizing problems (mean = 39.92, SD = 16.26). The family functioning and PYD levels of the surveyed adolescents were medium and above. In addition, the score for internalizing problems was lower than the norm (second-year junior high school students, mean = 44.51). Next, a Pearson correlation analysis was conducted on family functioning, PYD, and internalizing problems, and we found there was a significant correlation among the three (Table 1). Family functioning was positively correlated with PYD and negatively correlated with internalizing problems. PYD was negatively correlated with internalizing problems.

**Table 1:** Descriptive statistics and correlation analysis results of each variable (*n* = 11,865).

	**Mean**	**SD**	**1**	**2**
1. Family functioning	4.17	0.75		
2. Positive youth development	5.05	0.74	0.55^**^	
3. Internalizing problems	39.92	16.26	−0.47^**^	−0.51^**^

** *p < 0.01*.

In addition, a non-parametric test was used to determine the differences that migrant status made with all factors. We tested the data for normality using the Shapiro–Wilk method, with family functioning, PYD, and internalizing problems as dependent variables and migrant status as a factor and found that all *p*-values were <0.001, indicating that the data were not normally distributed. Thus, we used the Mann–Whitney *U*-test of two independent samples. Results showed that the average rank of migrant adolescents for family functioning (average rank = 5,413.81) was lower than that of local-born adolescents (average rank = 6,073.08), *z* = −8.599, *p* < 0.001; the average rank of migrant adolescents on PYD (average rank = 5,714.44) was lower than that of local-born adolescents (average rank = 5,991.97), *z* = −3.612, *p* < 0.001; the average rank of migrant adolescents on internalizing problems (average rank = 6,212.15) was lower than that of local-born adolescents (average rank = 5,857.69), *z* = −4.614, *p* < 0.001.

### Testing for the Mediation Model

To examine Hypothesis 1, model 4 of the PROCESS macro was used with age, gender, grade, and SES being controlled as the covariates. As shown in Table 2, family functioning was negatively associated with internalizing problems (*β* = −0.45, *p* < 0.001) in the absence of a mediator, whereas family functioning was positively correlated with PYD (*β* = 0.54, *p* < 0.001). In addition, when covariates and family functioning were controlled for, PYD was negatively associated with internalizing problems (*β* = −0.35, *p* < 0.001). When covariates and PYD were controlled for, the relationship between family functioning and internalizing problems was significantly negative (*β* = −0.26, *p* < 0.001). Finally, to examine the mediation model, the bias-corrected percentile bootstrap method was used. As shown in Table 3, the indirect effect of PYD was −0.19 (95% CI = −0.21 to −0.18); the empirical 95% CI did not contain zero, indicating that the mediation effect was significant. Furthermore, the mediation effect accounted for 42.33% of the total effect of the relationship between family functioning and internalizing problems. Thus, Hypothesis 1 was supported.

**Table 2:** Testing the mediation effects of family functioning on internalizing problems.

**Outcome variables**	**Independent variables**	*****β*****	**SE**	***t***	***p***	**LLCI**	**ULCI**
Internalizing problems	Constant	−0.30	0.06	−4.85	<0.001	−0.42	−0.18
	Family functioning	−0.45	0.01	−55.87	<0.001	−0.47	−0.43
	CO: age	0.06	0.60	0.10	0.924	−1.12	1.24
	CO: gender	0.08	0.02	4.94	<0.001	0.05	0.11
	CO: grade	0.08	0.01	5.63	<0.001	0.05	0.10
	CO: SES	−0.01	0.01	−4.17	<0.001	−0.02	−0.01
PYD	Constant	0.28	0.06	4.67	<0.001	0.16	0.39
	Family functioning	0.54	0.01	70.53	<0.001	0.53	0.56
	CO: age	0.54	0.57	0.04	0.349	−0.59	1.66
	CO: gender	−0.09	0.02	−6.11	<0.001	−0.12	−0.06
	CO: grade	−0.04	0.01	−3.56	<0.001	−0.06	−0.02
	CO: SES	0.01	0.01	4.18	<0.001	0.01	0.02
Internalizing problems	Constant	−0.20	0.06	−3.48	<0.001	−0.32	−0.09
	Family functioning	−0.26	0.01	−28.70	<0.001	−0.27	−0.24
	PYD	−0.35	0.01	−38.76	<0.001	−0.37	−0.34
	CO: age	0.25	0.57	0.43	0.664	−0.87	2.36
	CO: gender	0.05	0.02	3.07	<0.01	0.01	0.08
	CO: grade	0.06	0.01	4.71	<0.001	0.03	0.08
	CO: SES	−0.01	0.01	−2.93	<0.01	−0.02	−0.01

*n = 11,865; the *β* values are standardized coefficients. CO, control variable; LLCI, low limit of confidence interval; ULCI, upper limit of confidence interval; PYD, positive youth development*.

**Table 3:** Bootstrapping indirect effect and 95% confidence interval (CI) for the mediation model.

**Indirect path**	**Estimated effect**	**LLCI**	**ULCI**	**Ratio to total effect on internalizing problems**
Family functioning → PYD → internalizing problems	−0.19	−0.21	−0.18	42.33%

*n = 11,865. LLCI, low limit of confidence interval; ULCI, upper limit of confidence interval; PYD, positive youth development*.

### Testing for the Moderated Mediation Model

To examine Hypothesis 2 and Hypothesis 3, model 8 of the PROCESS macro was used, with age, gender, and SES controlled for as the covariates. The moderated mediation analyses are shown in Table 4. After controlling for demographic covariates, the mediator variable model indicated that family functioning was positively associated with PYD (*β* = 0.55, *p* < 0.001), and the interaction between family functioning and migrant status was not significant (*β* = −0.01, *p* = 0.699). Thus, Hypothesis 3 was not supported. As shown in the dependent variable model, family functioning was negatively correlated with internalizing problems (*β* = −0.15, *p* < 0.001), whereas the interaction between family functioning and migrant status was negatively correlated with internalizing problems (*β* = −0.06, *p* < 0.001). Therefore, migrant status moderated the relationship between family functioning and internalizing problems, and Hypothesis 2 was supported.

**Table 4:** Testing the moderated mediation effects of family functioning on internalizing problems.

	**Independent variables**	*****β*****	**SE**	***t***	***p***	**LLCI**	**ULCI**
**MEDIATOR VARIABLE MODEL**							
PYD	Constant	0.35	0.07	5.14	<0.001	0.21	0.48
	Family functioning	0.55	0.03	16.99	<0.001	0.49	0.62
	Migrant status	−0.04	0.02	−2.17	0.030	−0.08	−0.01
	Family functioning × migrant status	−0.01	0.02	−0.38	0.699	−0.04	0.03
	CO: age	−0.50	0.57	0.87	0.386	−0.62	1.62
	CO: gender	−0.09	0.01	−6.02	<0.001	−0.12	−0.06
	CO: grade	−0.05	0.01	−3.55	<0.001	−0.07	−0.02
	CO: SES	0.02	0.01	4.55	<0.001	−0.01	0.02
**DEPENDENT VARIABLE MODEL**							
Internalizing problems	Constant	−0.23	0.07	−3.45	<0.001	−0.36	−0.10
	Family functioning	−0.15	0.03	−4.58	<0.001	−0.22	−0.09
	PYD	−0.35	0.01	−38.76	<0.001	−0.37	−0.34
	Migrant status	0.02	0.02	0.87	0.386	−0.02	0.05
	Family functioning × migrant status	−0.06	0.02	−3.53	<0.001	−0.10	−0.03
	CO: age	0.26	0.57	0.46	0.648	−0.86	1.38
	CO: gender	0.05	0.02	3.05	<0.01	0.02	0.08
	CO: grade	0.06	0.01	4.72	<0.01	0.03	0.08
	CO: SES	−0.01	0.01	−3.03	<0.01	−0.01	−0.01

*n = 11,865, the beta values are standardized coefficients; CO, control variable; LLCI, low limit of confidence interval; ULCI, upper limit of confidence interval; PYD, positive youth development*.

**Figure 2:**
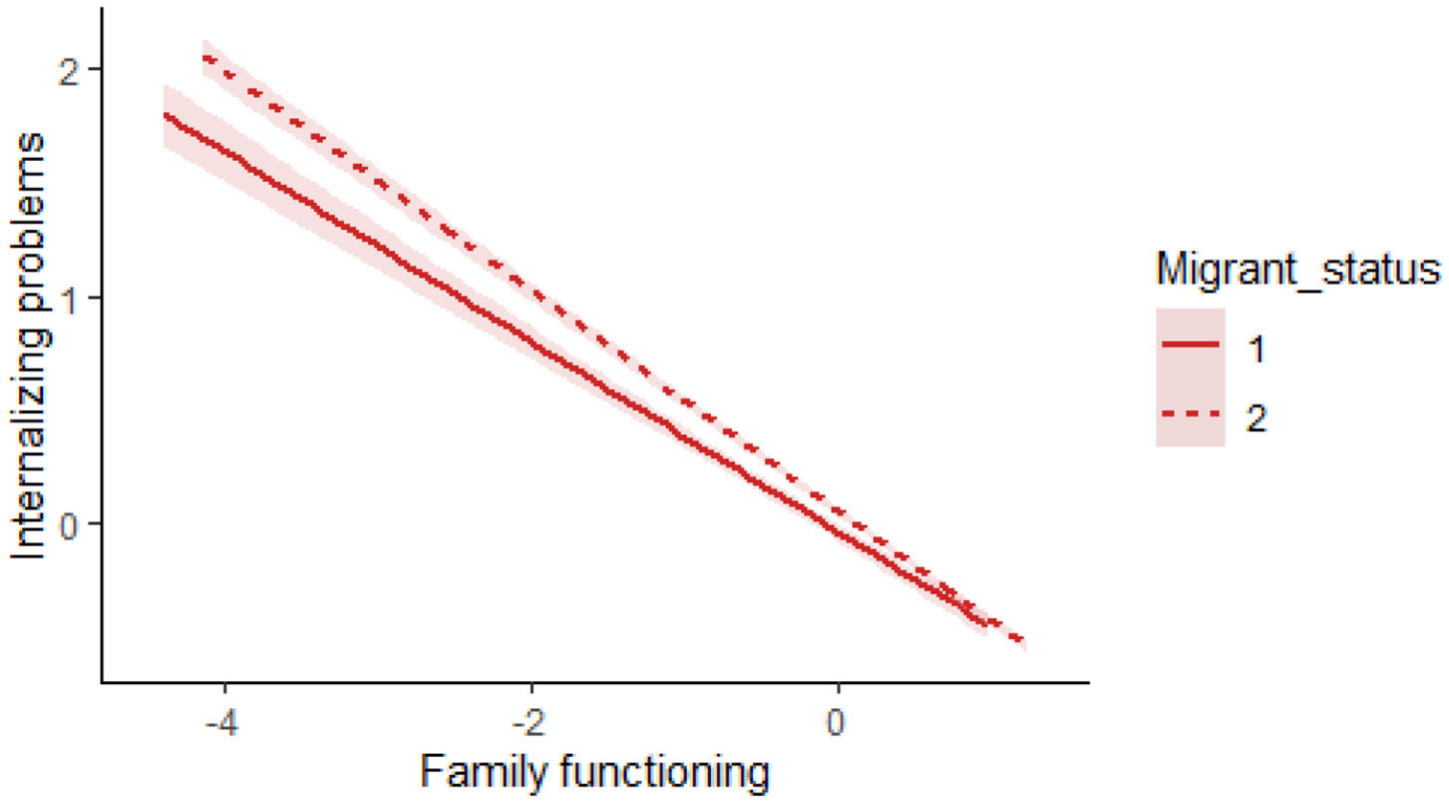
The moderating role of migrant status in the relationship between family functioning and internalizing problems. The solid line (migrant status is 1) indicates the group of migrant adolescents; the dotted line (migrant status is 2) indicates the group of local-born adolescents. The shadowed areas represent standard errors.

These results indicated that migrant status could moderate the relationships between family functioning and internalizing problems but could not moderate the relationships between family functioning and PYD. To more completely understand the moderating effects of migrant status, R Studio was used to create the image in Figure 2, which describes the relationships between family functioning and internalizing problems for two conditions of migrant status (i.e., migrant adolescents and local-born adolescents). In addition, the study further tested the conditional direct effect and indirect effect. As can be observed in Table 5, the effect of family functioning on internalizing problems was observed whether adolescents were migrants (*β* = −0.21, *p* < 0.001) or local-born (*β* = −0.28, *p* < 0.001). The 95% CI did not contain zero.

**Table 5:** Conditional direct effect of family functioning on internalizing problems for two migrant statuses.

**Classification**	**Direct effect**	**SE**	***t***	***p***	**95% CI**
Migrant adolescents	−0.21	0.02	−12.99	<0.001	[−0.25, −0.18]
Local-born adolescents	−0.28	0.01	−27.59	<0.001	[−0.30, −0.26]

*SE, standard error; CI, confidence interval. Confidence intervals that do not contain 0 are significant*.

## Discussion

Based on a large-scale sample, the present study examined a moderated mediation model and revealed the mechanism underlying the relationships between family functioning and internalizing problems among Chinese adolescents. The results showed that PYD significantly mediated the association between family functioning and internalizing problems in Chinese adolescents, and migrant status significantly moderated the relationship between family functioning and internalizing problems. These findings may provide theoretical and practical implications for the positive development of Chinese adolescents.

### The Mediating Role of Positive Youth Development

The study found that PYD partially accounted for the relationships between family functioning and internalizing problems among adolescents (H1 was supported), which was in line with previous studies (Catalano et al., 2002; Benson, 2006; Sun and Shek, 2013). In other words, family functioning not only can directly affect adolescents' internalizing problems but also can affect adolescents' internalizing problems through PYD attributes.

The development assets framework and the conservation of resources theory (Hobfoll, 2002; Benson et al., 2007) suggest that individuals' psychological resources may be weakened by a poor external environment, which may further lead to psychological stress. Such mental stress usually causes individuals to experience internalizing problems such as anxiety and depression (Scales et al., 2000; Hobfoll et al., 2003). By analogy, if the parent–child relationship is unsound, and family communication is lacking, this may negatively affect the formation of an adolescent's positive individual traits and psychological resources. Adolescents with deficient positive traits (e.g., resilience, emotional regulation ability) may not cope with adverse situations or stressful events, and these factors may ultimately increase the risk for the occurrence of internalizing problems (Lerner, 2007; Lougheed and Hollenstein, 2012). The findings of the present study indicate that improving PYD attributes may be a promising approach for reducing the risk of adolescent internalizing problems.

### The Moderating Role of Migrant Status

This study found that the effect of family functioning on internalizing problems was stronger among local-born adolescents than among migrant adolescents. Specifically, when family functioning was in good condition, there were minimal internalizing problems in either group, and there were no significant differences. However, when the family functioning was poor, internalizing problems increased significantly in both groups, and the problems were more serious in the group of local-born adolescents. These findings indicate that migrant status might serve as a buffer to alleviate the negative effects on adolescent development of unhealthy family functioning, which is in line with previous research (e.g., Hackett et al., 1991). The following factors may explain the findings.

The resiliency model suggests that in confronting the acculturation stress of migration, the migrant family gradually adjusts, which fosters resilience and personal strengths (e.g., toughness and grit; Greeff and Holtzkamp, 2007; Gui et al., 2012; Wu et al., 2014; Lan and Radin, 2020). Adolescents with high levels of resilience may be more proactive in adjusting their emotions and finding solutions to problems, which may, in turn, help prevent or reduce depression in migrant adolescents (Wingo et al., 2010; Masten and Tellegen, 2012; Ye et al., 2016). Moreover, researchers have suggested that aside from resilience, migrant youths may have some unobserved traits such as ambition or aspiration that enable them to cope with the transitional stress more independently and perhaps be less affected by poor family functioning (Georgiades et al., 2007; Chiswick et al., 2008; Sheidow et al., 2013). Several studies have reported that migrant youths outperform their local-born counterparts in school; they may be more ambitious and industrious than their native peers in order to achieve more academically (Fuligni, 1997; Motti-Stefanidi and Masten, 2013; Chen et al., 2019). These expectations of academic performance may buffer the influence on their mental health of poor family functioning. This study's findings indicated that migrant status may buffer the influence of poor family functioning on internalizing problems.

We found that migrant status had no significant moderating effect on the association of family functioning with PYD, which is similar to previous studies (e.g., Wissink et al., 2006). The findings indicate that regardless of whether adolescents are migrants, family functioning has an impact on their positive development, which reemphasizes the importance of family functioning and its general applicability to developing PYD attributes. Several factors may explain the non-significant moderating effect. First, almost all the migrant youths we investigated were attending public schools. Previous studies have demonstrated that migrant students in public schools exhibited greater psychosocial competencies (e.g., self-esteem) than migrant students in private schools, and they were not that different from their local peers (Li et al., 2008; Wang et al., 2010; Yuan, 2011). Our study confirmed previous research and found that the difference in the scores of PYD attributes between local-born and migrant children was fairly small (effect size = −0.05, Cohen *d* = −0.09). This leads to more similarity than difference in the effect of family functioning on PYD and thus leads to insignificant results. The second factor may be that the ratings of PYD attributes among both migrant and local-born adolescents were relatively positive (mean = 5.00 of 6.00 points for migrant adolescents vs. mean = 5.06 for local-born adolescents); the limited variation in PYD score may not be sufficient to be predicted by local and migrant children's family functioning (i.e., the ceiling effect; Weng et al., 2019; Chi et al., 2020).

### Limitation and Implications

There were several limitations to the present study. First, data from the cross-sectional design are unable to test the casual relationship that occurs over time between family functioning and internalizing problems in adolescents (Hu et al., 2009). To better verify the moderated mediation model, a future longitudinal study is required. Additionally, the direction between variables in our study was unidirectional (Figure 1), but the arrow direction between PYD and family functioning could also be bidirectional. Thus, cross-lagged longitudinal regression studies will be conducted to see if there are bidirectional relationships between the variables. Second, the measurement of family functioning in this study only included unilateral data from adolescents, and there is a lack of data from other paths, such as parents and teachers. Future research may fill this gap. Third, our study was conducted only in Shenzhen, and the generalizability of the findings is open to discussion. Thus, a nationwide survey is required in the future. Finally, the internal consistency of the questionnaires for PYD and internalizing problems in this study is too high, which may indicate that some items are testing the same question in different guises. This may also be the reason for the ceiling effect on PYD scores of migrant and local-born adolescents. Therefore, careful consideration is needed in the selection of scales for future research.

Although there are limitations, our findings have several theoretical and practical implications. First, the present study found a significant negative relationship between family functioning and internalizing problems, which indicates that adverse family circumstances may correlate with adolescent problem behaviors. Thus, special help is needed for adolescents in poorly functioning families to reduce their propensity for internalizing problems. For parents, better communication, decreasing conflict, and parental caring may be ensured to prevent children from internalizing problems. Second, a significant mediating role was found of PYD on the relationships between family functioning and internalizing problems. Thus, relevant courses or activities should be set up to cultivate and improve levels of PYD features to promote the adoption of positive strategies to cope with adverse situations and negative emotions. For policymakers, a PYD program could be carried out (e.g., Project P.A.T.H.S. in Hong Kong) to promote the positive development of youths (Shek and Sun, 2013). Third, we found a significant moderating role of migrant status on the relationships between family functioning and internalizing problems, which may contribute to a better understanding of the mechanism behind these correlations in adolescents. Specifically, migrant status could buffer the influence of poor family functioning on internalizing problems among adolescents in the study. The results make efforts important for reducing the effects of negative stereotypes about migrant adolescents and, most importantly, to help them enhance self-efficacy, resist adverse environments, and reduce problem behaviors.

## Data Availability Statement

The datasets presented in this article are not readily available because the project is building another project based on this dataset. Requests to access the datasets should be directed to xinlichi@126.com.

## Ethics Statement

Recruitment and data collection procedures were approved by the Human Research Ethics Committee (No:2020005) of Shenzhen University. Written informed consent to participate in this study was provided by the participants' legal guardian/next of kin.

## Author Contributions

QW and XC conceived the study. QW conducted all data collection work. SP conducted statistical analysis and wrote the manuscript. XC critically reviewed the manuscript. XC and SP revised the manuscript. All authors contributed to and have approved the final manuscript.

## Conflict of Interest

The authors declare that the research was conducted in the absence of any commercial or financial relationships that could be construed as a potential conflict of interest.
